# Case Report: Nephrotic syndrome as the primary manifestation of Alport syndrome in a Chinese pediatric patient

**DOI:** 10.3389/fped.2024.1518553

**Published:** 2025-01-08

**Authors:** Yue Song, Yifei Li, Liqun Lu, Changqiang Yang, Jing Lu

**Affiliations:** ^1^ Department of Pediatrics, The First Affiliated Hospital of Chengdu Medical College, Chengdu, Sichuan, China; ^2^ Department of Pediatrics, West China Second University Hospital, Sichuan University, Chengdu, China; ^3^ Department of Cardiology, The First Affiliated Hospital of Chengdu Medical College, Chengdu, Sichuan, China

**Keywords:** Alport syndrome, nephrotic syndrome, proteinuria, hematuria, *COL4A3*

## Abstract

**Background:**

Alport syndrome (AS) is a genetically heterogeneous disorder resulting from variants in genes coding for the alpha-3/4/5 chains of Collagen IV, leading to defective basement membranes in the kidney, cochlea, and eye. The clinical manifestations of AS vary in patients. Cases of childhood AS caused by *COL4A3* presenting primarily with nephrotic syndrome (NS) are rarely reported. Here, we report a pediatric case presenting initially with NS attributed to AS caused by *COL4A3*.

**Case presentation:**

An 11-year-old boy presented with hematuria and nephrotic range proteinuria. After excluding secondary causes, primary NS was considered. He was administered with prednisone (60 mg/day). The patient had not responded to treatment by the end of 4 weeks, so he was diagnosed with steroid-resistant NS. A renal biopsy showed granular and vacuolar degeneration of renal tubular epithelial cells, multifocal foam cell infiltration in the renal interstitium, and immunofluorescence indicated the absence of α3, α4, and α5 expression in the glomerular and tubular basement membrane, while Bowman's capsule expression was normal. Electron microscopy ultrastructural suggested variable basement membrane thickness, and partial tearing and web-like structures. Genetic testing revealed a heterozygous *COL4A3* missense mutation c.3210 (exon 37)G>A(NM:000091). These findings are consistent with the diagnosis of AS. Prednisone was gradually tapered and enalapril maleate was initiated.

**Conclusion:**

We have described a pediatric case of AS featuring NS as its primary manifestation. It is important to consider AS to be a diagnosis or differential diagnosis in patients who have NS with hematuria or steroid resistance.

## Introduction

Alport syndrome (AS) is an inherited kidney disorder resulting from variants in genes coding for alpha-3/4/5 chains of Collagen IV, which results in defective basement membranes in the kidney, cochlea, and eye (1). Disruption or alteration of these collagens leads to a breakdown of their structure and function, which display various clinical manifestations such as hematuria, proteinuria, progressive renal failure, and retinal flecks (2). Notably, nephrotic syndrome (NS) can also be an atypical manifestation in cases of AS, and distinguishing AS from NS can be challenging.

Children with AS who initially presented as NS cases have rarely been reported (3), especially in children with AS caused by *COL4A3*. Massive proteinuria is associated with poor renal prognosis; therefore, the early diagnosis and treatment of AS are of great importance in delaying the onset of end-stage renal disease (ESRD) in affected children. Here, we report a case of AS in an 11-year-old boy who presented with NS, with no known family history of renal disease, who was diagnosed through pathological investigations and genetic testing. By reporting this case, we hope to raise awareness of the disease and highlight the importance of renal pathology and genetic testing in diagnosing AS, offering valuable insights for clinicians and researchers.

## Case presentation

An 11-year-old Chinese boy was admitted to the pediatric department of the First Affiliated Hospital of Chengdu Medical College for vomiting and abdominal pain for 1 day. The pain, occurring sporadically in the whole abdominal area, was mild and was accompanied by fever and diarrhea. He also consulted for nephrotic range proteinuria and hematuria. His parents and younger brother had no history of kidney disease. He is not from a consanguineous family.

The patient's vital signs were normal, with a body temperature of 36.6°C, pulse of 86 beats/min, and respiratory rate of 20 breaths/min. His body mass was 30 kg (25th percentile), stature was 136 cm (P10), and his blood pressure was 116/66 mmHg (90th–95th percentile). Physical examination was unremarkable with no edema. Laboratory investigations showed elevated serum urea nitrogen (10.64 mmol/L), normal creatinine (Cr; 42 µmol/L), decreased albumin (24.1 g/L), elevated total cholesterol (12.18 mmol/L), elevated fibrin degradation products (2.45 µg/mL), and 3.39 g/24 h 24-h urine protein quantification. The routine blood test revealed 26.86 × 10^9^/L leukocytes, 84.5% neutrophils, 130 g/L hemoglobin, and C-reactive protein 15 mg/L. Urinalysis showed proteinuria (3+) and hematuria (2+). Tests for anti-nuclear, anti-cardiolipin, antineutrophil cytoplasmic, and anti-glomerular basement membrane antibodies, as well as hepatitis B and C serology and rheumatoid factor, were all negative. In addition, complement components C3 and C4, as well as thyroid function, were within normal limits. The norovirus antigen test was positive, and stool routine and culture were normal. Abdominal CT showed a small amount of pelvic effusion. Renal ultrasonography revealed normal-sized kidneys. Based on these findings, secondary causes of NS were excluded and the patient was diagnosed with primary NS. NS with microscopic hematuria is an indicator for renal biopsy. His parents strongly preferred prednisone therapy, viewing the kidney biopsy as invasive. He was therefore administered cefoperazone sulbactam, oral steroid medication (2 mg/kg), fluid therapy, and gastric protection treatments. The patient was discharged after the abdominal pain and vomiting subsided.

**Figure 1:**
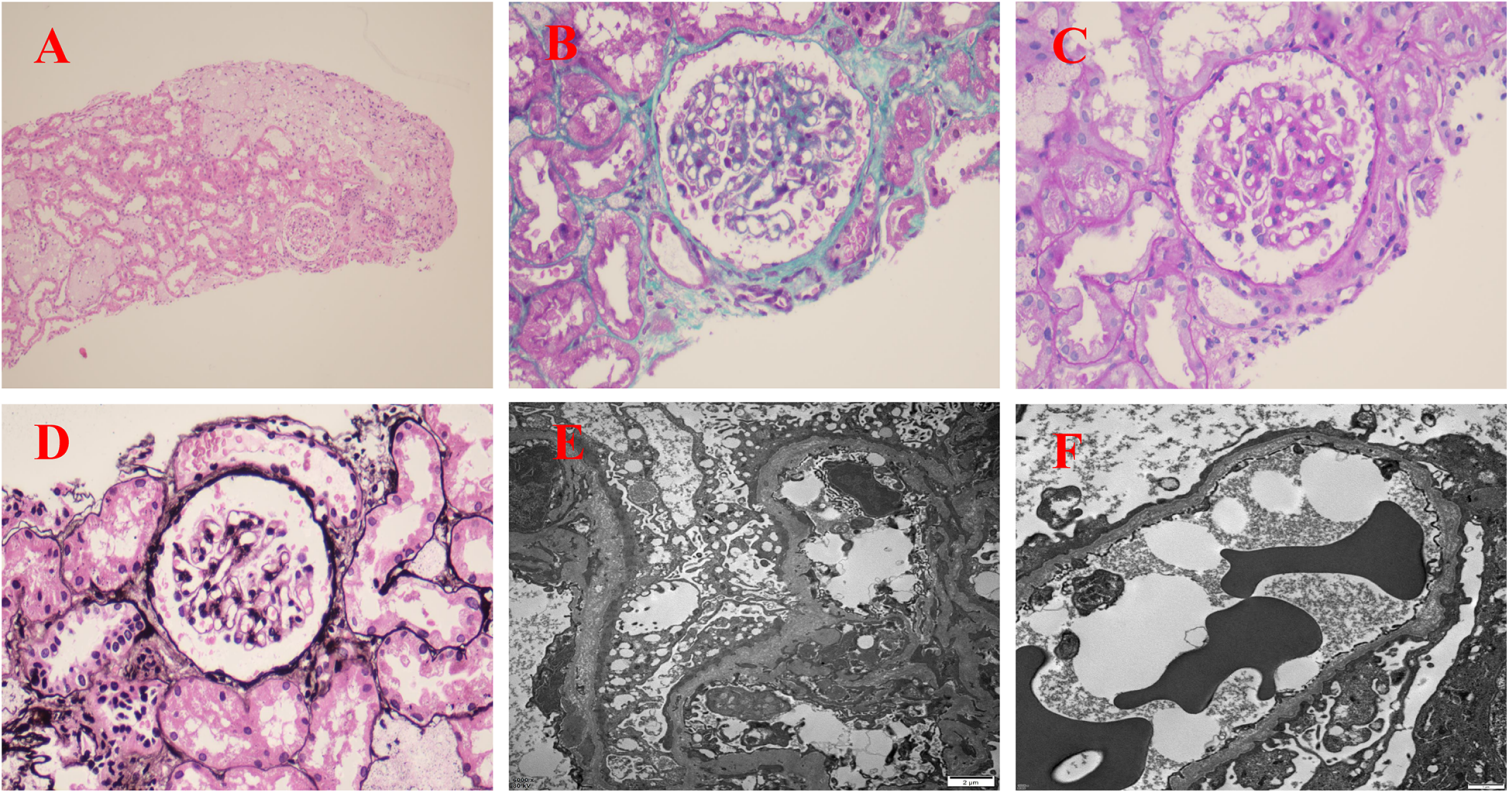
Renal pathological examination: **(A)** H&E staining (×100); **(B)** Masson staining (×400); **(C)** Periodic acid-Schiff (PAS) staining (×400); **(D)** Periodic acid-silver metheramine (PASM) staining (×400); **(E,F)** electron microscope image (×5,000). **(A–D)** Renal pathology revealed mild segmental proliferation of mesangial cells and matrix within the glomeruli, granular and vacuolar degeneration of renal tubular epithelial cells, a few renal tubules with luminal dilation accompanied by segmental epithelial cell detachment, occasional renal tubule atrophy, and multifocal foam cell infiltration in the renal interstitium. **(E**,**F)** The basement membrane varied in thickness, with thickening of the dense layer, some parts appeared torn or reticulated, and partial fusion of foot processes.

**Figure 2:**
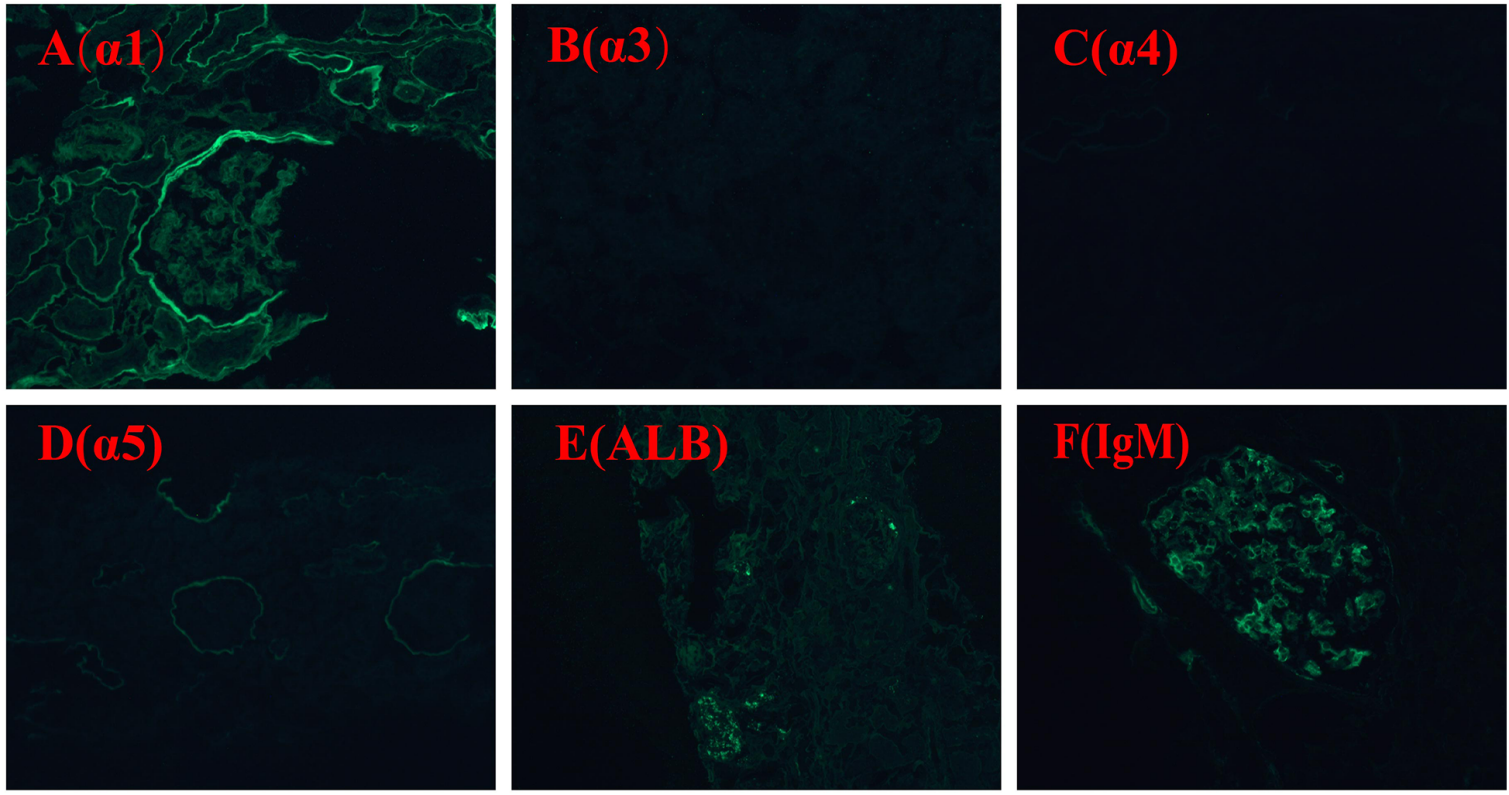
Immunofluorescence staining α1, α3, α4, α5, ALB, IgM: α1 positive control is normal; expression of α3, α4, and α5 in glomerular basement membrane and tubular basement membrane is absent, expression in Bowman's capsule is normal. ALB (−); IgM (+).

**Figure 3:**
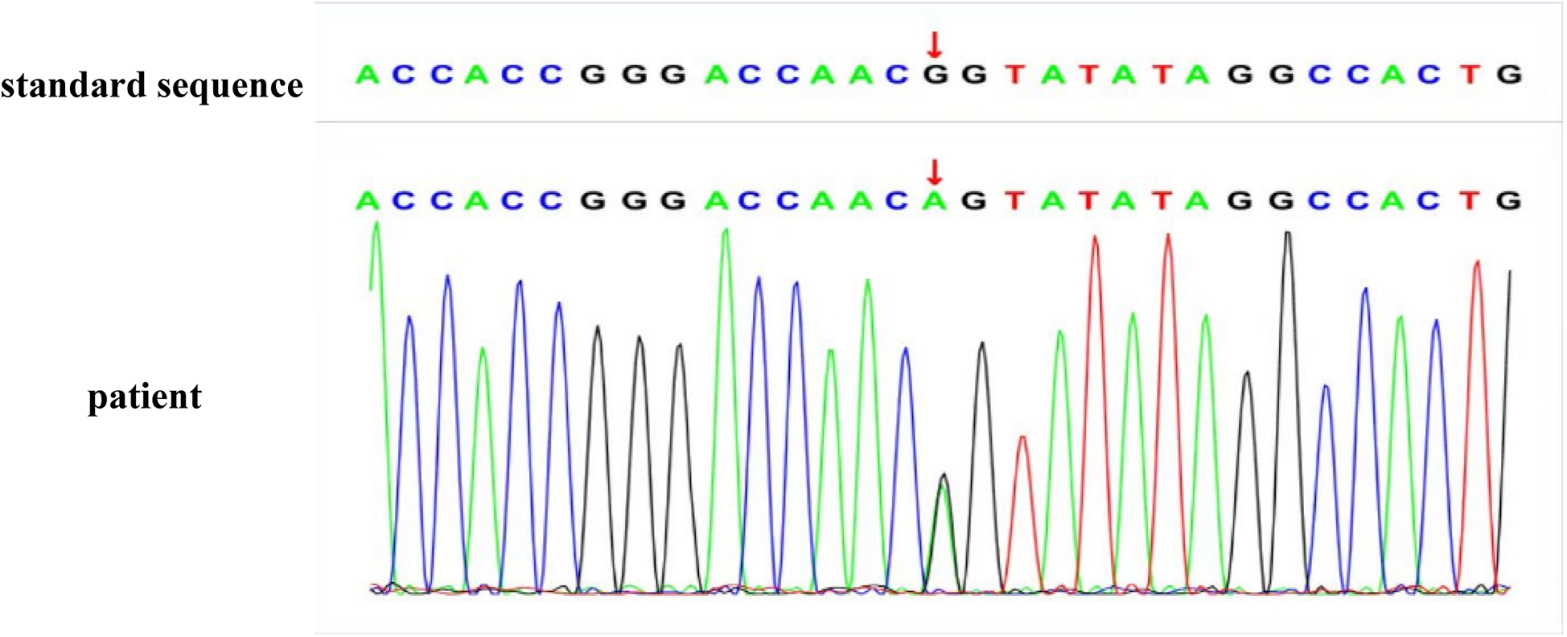
The genetic sequence of the patient: the patient has a heterozygous mutation at the gene locus.

Due to persistent proteinuria and hematuria, the child underwent a renal pathological and genetic test at the West China Second Hospital of Sichuan University. Renal pathology revealed mild segmental proliferation of mesangial cells and matrix within the glomeruli, granular and vacuolar degeneration of renal tubular epithelial cells, a few renal tubules with luminal dilation accompanied by segmental epithelial cell detachment, occasional renal tubule atrophy, and multifocal foam cell infiltration in the renal interstitium (Figure 1). Immunofluorescence indicated normal α1 positive control, as well as absent expression of α3, α4, and α5 in the glomerular basement membrane and tubular basement membrane, with normal expression in Bowman's capsule (Figure 2). Electron microscopy ultrastructure revealed irregular basement membrane thickness, thickening of the dense layer of the basement membrane, areas of partial tearing with a web-like appearance, and partial foot process fusion. No electron-dense material deposition was observed. These findings are considered indicative of AS (Figure 1). Subsequent audiometry revealed mild sensorineural hearing loss in both ears. Fundus examination indicated retinal depigmentation changes and refractive error in both eyes. Genetic testing confirmed the presence of a heterozygous *COL4A3* missense mutation c.3210 (exon 37) G>A (NM:000091) (Figure 3). The patient’s father and younger brother were asymptomatic and his mother was otherwise healthy. The urinalysis of the patient's parents and younger brother was normal. After the confirmation of AS, the child was started on oral enalapril maleate and the oral steroid medication was gradually tapered. The patient was regularly monitored through outpatient clinic follow-ups. A 6-month follow-up revealed persistent nephrotic range proteinuria and hematuria, while Cr and blood urea nitrogen levels were normal.

## Discussion

The child in this case had persistent glomerular hematuria with nephrotic range proteinuria, accompanied by hearing loss and eye changes. Electron microscopy indicated tearing and reticular changes in the dense layer of the basement membrane. Immunofluorescence staining of type IV collagen α3, α4, and α5 chains in the renal tissue basement membrane was abnormal, and genetic testing showed a pathogenic variation in the *COL4A3* gene. According to the 2023 Expert Recommendations for the Diagnosis and Treatment of AS in China, the AS diagnostic criteria (4), the diagnosis of AS caused by the pathogenic variation in the *COL4A3* gene was confirmed.

AS caused by pathogenic variations in the *COL4A3* gene can be classified into autosomal dominant AS, autosomal recessive AS, and digenic AS. Patients with AS due to pathogenic variations in the *COL4A3* gene show various manifestations, which may include isolated hematuria, hematuria with proteinuria, reduced renal function, hearing loss, and ocular abnormalities. Renal pathology may reveal focal segmental glomerulosclerosis, thin glomerular basement membrane, and typical AS changes. In our case, the child's initial manifestation was NS. Although proteinuria may be an initial symptom of AS, cases presenting initially with NS caused by *COL4A3* are rare (5–7). A systematic review of 148 patients with autosomal recessive AS patients found that only 27 patients developed nephrotic range proteinuria during the course of the disease, with the median age of genetic diagnosis for autosomal recessive AS being 20 years, and 62% of patients developed ESRD at a median age of 21 years (8). Weber et al. (9–11) found that 100% of 37 patients with autosomal dominant AS due to pathogenic variations in the *COL4A3* gene had hematuria, approximately 14.8% had proteinuria, and approximately 13.3% developed ESRD at the age 34–52 years. In this case, the child initially presented with NS and hematuria, and due to prednisone resistance, underwent renal biopsy and genetic testing, ultimately confirming AS. Massive proteinuria is associated with a poor renal prognosis, making the early diagnosis and treatment of AS significant in delaying the onset of ESRD. It is also noteworthy that AS can be concurrent with other renal diseases, such as lipoprotein glomerulopathy, IgA nephropathy, thin basement membrane nephropathy, and C3 glomerulonephritis (12–14). It is important to consider AS as a diagnosis or differential diagnosis, especially in patients who have NS with hematuria or steroid resistance. In addition, the timing of renal biopsy holds significant clinical importance. Current guidelines advocate for performing a renal biopsy in pediatric patients aged over 12 years, primarily due to the heightened incidence of focal segmental glomerulosclerosis and other glomerulopathies, including membranous nephropathy and membranoproliferative glomerulonephritis (15, 16). Certainly, early renal biopsy and genetic analysis are imperative for directing therapeutic strategies and prognostic assessments in pediatric patients, thereby effectively circumventing the administration of corticosteroids and mitigating associated adverse effects. However, the optimal age for performing a renal biopsy at the initial presentation of NS remains contentious.

The proband, along with his father and younger brother, harbored the identical genetic mutation; however, clinical manifestations, such as hematuria and proteinuria, were observed exclusively in the proband. Neither the father nor the younger brother exhibited any related phenotypic symptoms, and the mother was devoid of the pathogenic allele. Although the mutation patterns were heterozygous, the mode of inheritance could not be clarified. Mutations previously thought to be autosomal recessively inherited can also be inherited autosomally dominantly (5). We speculated that the proband may have a compound heterozygous mutation, which might have another variant of the maternal allele but could not detect it because it may have been an intronic variant (5). Moreover, the observed clinical variability cannot be attributed exclusively to genetic mutations and may be associated with variances in penetrance, environmental influences, or other unidentified genetic discrepancies. Epigenetic mechanisms such as DNA methylation, histone modifications, chromatin remodeling, and regulation by non-coding RNAs may contribute to this heterogeneity (17). This phenomenon is a reminder that AS caused by *COL4A3* mutations may present early in patients when their family members are asymptomatic.

Currently, there is no specific treatment for AS. In our case, enalapril maleate was administered. This is because angiotensin-converting enzyme inhibitors (ACEI) can reduce proteinuria in AS patients caused by pathological mutations in the *COL4A3* gene and prolong the time to progression to ESRD (18). Moreover, distinct genetic profiles of AS may necessitate varied therapeutic strategies: individuals with autosomal recessive AS resulting from pathogenic mutations in the *COL4A3* gene or AS with biallelic mutations in the *COL4A3* and *COL4A4* genes should initiate ACEI therapy at the onset of hematuria; whereas those with autosomal dominant AS attributed to pathogenic mutations in the *COL4A3* gene or AS with monoallelic mutations in the *COL4A3* and *COL4A4* genes should commence ACEI therapy when the urine microalbumin/creatinine ratio exceeds 30 mg/g (19). In addition, several basic or clinical studies (20–24) have suggested that cyclosporine A, bardoxolone, microRNA-21, sodium-glucose cotransporter 2 inhibitors, hydroxypropyl-β-cyclodextrin, and histone deacetylase inhibitors may also be new treatment options for AS. For AS patients who progress to ESRD, long-term dialysis and kidney transplantation are treatment methods to prolong life, with kidney transplantation being the more ideal renal replacement therapy (25).

We have described a pediatric case of AS featuring NS as its primary manifestation. It is important to consider AS as a diagnosis or differential diagnosis in patients who have NS with hematuria or steroid resistance. Renal pathology and genetic testing may be crucial to diagnose the disease early and facilitate timely management.

## Data Availability

All relevant data is contained within the article: The original contributions presented in the study are included in the article, further inquiries can be directed to the corresponding author, Jing Lu, lujing_95@163.com.
